# Dynamics of tertiary lymphoid structures and immune cross talk in early versus advanced colorectal cancer: potential implications for immunotherapy

**DOI:** 10.1007/s00262-025-04027-x

**Published:** 2025-04-26

**Authors:** Zixu Chen, Bang Hu, Keyu Cai, Han Gao, Zhenyu Xian, Shuang Zhang, Zhen Fang, Qian Zhou, Donglin Ren, Qi Zou

**Affiliations:** 1 School of Basic Medical Sciences, Shandong Provincial Hospital, Shandong University, Jinan, 250012 Shandong People’s Republic of China; 2 Guangdong Institute of Gastroenterology, Guangdong Provincial Key Laboratory of Colorectal and Pelvic Floor Disease, The Sixth Affiliated Hospital, Sun Yat-Sen University, Guangzhou, 510655 Guangdong People’s Republic of China; 3 Department of Colorectal Surgery, Department of General Surgery, The Sixth Affiliated Hospital, Sun Yat-Sen University, Guangzhou, 510655 Guangdong People’s Republic of China; 4 Biomedical Innovation Center, The Sixth Affiliated Hospital, Sun Yat-Sen University, Guangzhou, 510655 Guangdong People’s Republic of China; 5 Department of Emergency Surgery, The Affiliated Hospital of Qingdao University, Qingdao, 266000 Shandong People’s Republic of China; 6 Department of Gastrointestinal Surgery, Shandong Provincial Hospital Affiliated to Shandong First Medical University, Jinan, 250000 Shandong People’s Republic of China; 7 Department of Gastrointestinal Surgery, Shenshan Medical Center, Sun Yat-Sen Memorial Hospital, Sun Yat-Sen University, Shanwei, 516600 People’s Republic of China

**Keywords:** Tumor microenvironment, Immunotherapy, Tertiary lymphoid structure, Colorectal cancer

## Abstract

**Background:**

Irrespective of microsatellite status, immune checkpoint inhibitor therapy shows superior efficacy in early-stage colorectal cancer (CRC) compared to advanced cases. The distinctions of the tumor microenvironment (TME) and tertiary lymphoid structure (TLS) between early- and advanced-stage CRC may represent a critical factor, yet remain incompletely elucidated.

**Methods:**

We comprehensively analyzed single-cell RNA sequencing data, bulk RNA transcription data and pathological tissue data to investigate the dynamic changes in the TME. The features of TLS in early- and advanced-stage tumors and their potential impact on immunotherapy were explored using three in-house cohorts.

**Results:**

We provided single-cell fine maps of the immune landscape in early and advanced CRC. Significant functional differences were identified in CD4 + Tfh and BGC cells between early and advanced CRC. We revealed CXCL13 expression on CD8 + Tex cells, along with CD40–CD40L interactions between CD4 + Tfh and BGC cells, could be key regulators of TLS functionality and subsequently affect the response to immunotherapy.

**Conclusions:**

Our research shed light on the multilayered immune dysfunction in advanced CRC and elucidates the alterations in the TLS during the progression of CRC, providing insights for functional studies and the exploration of potential target in advanced CRC.

**Supplementary Information:**

The online version contains supplementary material available at 10.1007/s00262-025-04027-x.

## Background

Immune checkpoint inhibitor (ICI) therapy has exhibited a promising role in the treatment of mismatch repair-deficient and microsatellite instability-high (dMMR/MSI-H) colorectal cancer (CRC). However, a certain subpopulation still persists as non-responders to ICI therapy, which represents a huge heterogeneity even among dMMR/MSI-H CRC, especially in the subset with advanced stages. In early-stage mismatch repair-proficient and microsatellite-stable (pMMR/MSS) CRC, the pathological response rate of neoadjuvant ICI treatment has exceeded an impressive one-fourth [1]. Similarly, for a few cancer types that are considered to benefit from ICI therapy, the response rate in the early stage appears to be more encouraging than in advanced stages [2, 3]. This indicates that irrespective of the microsatellite status, there is a pronounced disparity in the efficacy of early- and advanced-stage CRC to ICI therapy, and treating certain subset of advanced-stage tumors poses a challenge.

The composition and functional status of the tumor microenvironment (TME) in CRC are considered crucial parameters determining the efficacy of ICI therapy [4, 5]. Throughout the developmental trajectory from early to advanced stages of tumor, substantial temporal alterations occur within the TME [6]. As the tumor progresses, a conspicuous decreased infiltration of some crucial immune subgroups may be observed, such as cytotoxic CD8 + T cells [7]. Furthermore, the immunosuppressive microenvironment of advanced-stage tumors may lead to time-dependent exhaustion of T cells, thus weakening the response to ICI therapy [8]. The dynamic changes of the cytokine CXCL13 at each stage of the tumor may affect the infiltration and function of Tfh cells [9]. The functional alterations of these cells may impact the status of the tertiary lymphoid structure (TLS), including its maturity. Simultaneously, the density of TLS has been reported to be associated with ICI therapy [10]. The varying density and functionality of these immune components, in conjunction with tumor staging, may result in significant differences in treatment strategies between early- and advanced-stage tumors.

A comprehensive understanding of the dynamic changes of TLS and the functional alterations of its cellular components in early- and advanced-stage CRC is lacking, hindering innovation in immunotherapy for advanced CRC. Single-cell RNA sequencing enables high-resolution characterization of the complex TME, revealing the proportions and functions of distinct cell subpopulations, exploring cell-to-cell interactions and dynamic evolution.

Here, we conducted a thorough analysis of the TME in early and advanced CRC by integrating multiple single-cell sequencing-based datasets, irrespective of the microsatellite status. Our findings highlighted notable variations in multiple cell subpopulations and TLSs that might be correlated with tumor immunotherapy, which may provide a new perspective to unravel the mechanisms for immune evasion in advanced-stage CRC and improve the response of ICI therapy.

## Materials and methods

### Data retrieval and preprocessing

The bulk RNA-seq data and corresponding survival and clinical information of CRC patients from The Cancer Genome Atlas (TCGA) (TCGA CRC cohort, including TCGA colon adenocarcinoma and rectum adenocarcinoma cohort) were obtained using the “TCGAbiolinks” R package (version 2.28.3). Samples with incomplete survival information and clinical information, and those with an overall survival (OS) of less than 30 days, were excluded. Patients with stage I/II (according to the American Joint Committee on Cancer TNM staging system) of CRC were considered as early stage while those with stage III/IV of CRC were grouped as advanced stage in TCGA CRC cohort.

The scRNA-seq data of CRC were downloaded from the Gene Expression Omnibus (GEO) database, containing scRNA-seq of pharmaceutically untreated primary tumor specimens of 26 CRC patients from six GEO datasets (GSE110009, GSE200997, GSE188711, GSE144735, GSE132465, GSE161277). Patients were classified as having early-stage (postoperative T1/2, N0, M0) or advanced-stage (postoperative T3/4, any N, M1 or T4, any N, any M) tumor on postoperative pathology. The core cells of scRNA-seq data were obtained by filtering scRNA-seq based on the total unique molecular identifier (UMI) counts, number of detected genes and proportion of mitochondrial gene counts per cell. Briefly, ineligible cells include genes that can only be detected in 3 or fewer cells and low-quality cells with excessively high or low gene number detected, or cells with low complexity, identified as those with log10GenesPerUMI (the gene numbers of per UMI) less than 0.8 and cells with the mitochondrial gene ratio of greater than 20% would be excluded, yielding a filtered count matrix with 42,503 cells and 30,260 genes for downstream analysis.

### scRNA-seq data analysis

After quality control, gene expression of filtered cells was normalized and scaled by regressing out the percentage of mitochondrial genes with the “SCTransform” function (version 0.3.5). We removed the batch effect across different datasets by using an “Anchor” method in the R package “Seurat” (version 4.3.0.1). Dimensional reduction and visualization were performed via principal component analysis and uniform manifold approximation and projection (UMAP): using the top 40 principal components for whole-cell types. Lastly, the “FindClusters” function was adopted for cell clustering, and the resolution was set to 0.8. Certain known canonical markers (T cells: *CD3D*, *CD3E*, *CD3G*, *CD40LG*; B cells: *CD79A*, *CD79B*, *MS4A1*, *MZB1*; NK cells: *KLRD1*, *KLRF1*, *NKG7*; myeloid cells: *CD68, CD86*, *MNDA*; epithelial cells: *EPCAM*, *CLDN3*; fibroblast: *LUM*, *COL1A1*, *DCN*; endothelial cells: *PECAM1*, *FLT1*, *RRAMP2*) were used to annotate each cluster.

Using T cell markers (*CD3D*, *CD3E*, *CD3G*), we identified a total of 15,282 cells, which were annotated as T cells. Then CD8 + T and CD4 + T cells were isolated based on average expression of CD8 genes (*CD8A*, *CD8B*) and CD4 (average expression of CD8 genes ≥ average expression of *CD4*, CD8 + T cells; average expression of CD8 genes < average expression of *CD4*, CD4 + T cells). The CD8 + and CD4 + T cells were processed separated in downstream clustering and analysis.

Endothelial cells, fibroblast and immune cells were subset from the raw expression matrix and the same preprocessing steps, and further clustering was performed to obtain the subpopulation structures. The subclustering of all these cells was performed for using all combinations of principal components (running from 15 to 35) and resolution parameters (0.2, 0.3,0.4, 0.5, 0.6, 0.7 and 0.8).

To character each cluster, we utilized the “FindAllMarkers” function of the Seurat package, which identified markers using log fold change (FC) of the average expression, using default parameters. Genes that met these criteria were considered marker genes: FC > 0.25 and pct > 0.25 (the percentage of cells where the feature is detected in either group), and the mitochondrial genes and ribosomal genes were excluded. Furtherly, the cell identity of each cluster was determined by comparing these marker genes through the “FindAllMarkers” function and known cell markers from literature references. The marker gene lists are shown in Table S1.

### Defining cell state scores

We used cell scores to evaluate the degree to which individual cells expressed predefined gene sets associated with functional states, including cytotoxicity, exhaustion, naïveness, effector memory, stress response, proliferation, activation and inflammatory signatures. The “AddModuleScore” function in Seurat was used to implement the scoring with default settings. Signature scores were defined by the average expression of published signature gene of the gene lists [11–16]. All gene lists defining these scores, along with their original references, are provided in Table S2.

### Gene functional annotation

To compare the functional profiles of CD4 + Tfh cells and B cells from early- and advanced-stage CRC, we used “clusterProfiler” R package (version 4.8.2) for Gene Ontology (GO) pathway analysis with differentially expressed genes (DEGs).

To generate the gene set of CD4 + germinal center (GC) Tfh signature for gene set enriched analysis (GSEA), we analyzed the microarray data from GSE accession number GSE50391 previously published [17]. Genes with the adjusted p value < 0.5 and the value of logFC more than 0.8 were selected when comparing the GC Tfh cells to the Tfh cells, and constituted as the gene set for subsequent GSEA analysis in CD4 + Tfh cells using the “fgsea” R package (version 1.26.0) (nperm = 1000).

### Cell–cell communication

To infer the cell–cell interactions and to identify the mechanism by which the molecules communicate at a single-cell resolution, the R package “CellChat” (version 1.6.1) was applied. All databases curated in CellChatDB.human database including the “Secreted Signaling,” “Cell–Cell contact” and “ECM-Receptor” were included. Briefly, we followed the official workflow and created the CellChat object. Using the “aggregateNet” function in CellChat, the aggregated cell–cell communication network was calculated, and the signaling from two groups was visualized. Outgoing or incoming signals of certain cell types were recognized using the function “netAnalysis_signalingRole_heatmap.” The most significant ligand–receptor pairs that mediate the cell–cell interaction changes across comparisons by using the “netVisual_bubble” function in CellChat.

### Cell differentiation trajectory analysis

To explore potential differentiation routines of CD8 + and CD4 + T cells, we performed trajectory analysis via the Slingshot package (version 2.6.0). The Seurat object was transformed into a SingleCellExperiment object, and Slingshot trajectory analysis was performed using Seurat clustering information and UMAP dimensionality reduction. The calculated trajectories were overlaid into the UMAP embeddings. Genes that varied across the Slingshot trajectories were investigated with tradeSeq R packages (version 1.13.04).

### Patient cohorts

The three cohorts consist of CRC patients surgically treated and pathologically diagnosed at the Sixth Affiliated Hospital of Sun Yat-sen University. Specifically, SYSUSH cohort consists of 170 CRC patients diagnosed between 2008 and 2012, with the formalin-fixed and paraffin-embedded slides available for CD8 IHC staining. SYSUSH Cohort 2 includes 76 CRC patients from the same period with H&E-stained slides available for TLS analysis. SYSUSH cohort 3 comprises 15 cases of advanced (stage III or IV) rectal cancer treated with neoadjuvant PD-1 antibody therapy between 2020 and 2023, with immunotherapy indications of dMMR/MSI-H. The clinicopathological information of these patients in Cohort 3 is listed in Table S3. Responder was defined as a partial response or complete response.

### Immunohistochemistry (IHC) assessment of CD8 + T cell infiltration

The formalin-fixed and paraffin-embedded tumor blocks were cut into 4 µm sections. IHC with monoclonal anti-CD8 antibody (DAKO) was conducted. CD8 + T cell infiltration was assessed by two independent pathologists based on five representative fields of each slide. The procedure was described in detail previously [8].

### Counting TLS based on hematoxylin–eosin (H&E) staining

One pathologist evaluated the linear distribution of TLS along the tumor’s invasive margin on H&E-stained slides, calculating the average number of TLS per 10 mm by dividing the TLS count by the length of the invasive margin on each slide.

#### Multiple fluorescence IHC (mfIHC) for tumor-infiltrating leukocytes and TLSs in CRC

Tumor-infiltrating leukocytes (including T cells, B cells and DC) and TLSs were detected using mfIHC staining in the two groups of CRC samples. Antibody kits used included CD4 (ab133616), CD8 (ab237709), CD20 (ab64088), CD21 (ab75985), CD23 (ab92495), CXCR5 (ab46218) and CXCL13 (10,927-1-AP). mfIHC was performed using Opal 6-Color Manual IHC kit (Panovue Biotechnology) according to the manufacturer’s protocol. Briefly, different primary antibodies were sequentially applied, followed by horseradish peroxidase-conjugated secondary antibody incubation and tyramide signal amplification (TSA). The slides were microwave heat-treated after each TSA round. Nuclei were stained with 4′-6-diamidino-2-phenylindole (DAPI) after all the human antigens had been labeled. Scanning and analysis of mfIHC slides were performed on a PhenoImager HT System (Akoya Biosciences, Waltham, USA) or the KF-FL-400 digital pathology slide scanner (KFBIO, Ningbo, China). Specifically, we screened mature TLS and immature TLS on the basis of cellular marker composition (CD4, CD8, CD20, CD21 and CD23) [18]. Mature TLSs were organized lymphoid structures containing B lymphocytes admixed to CD23 + follicular dendritic cells.

#### Western blot analysis

Tumor tissues were lysed in RIPA lysis buffer supplemented with a proteasome inhibitor (Beyotime). Total proteins were separated by 10% sodium dodecyl sulfate polyacrylamide gel electrophoresis and then transferred to a polyvinylidene fluoride membrane (Millipore). After blocking in 5% nonfat milk for 1 h, the membranes were incubated with antibodies specific to β-actin (1:1500, Abclonal), HSPA1A (1:800, Abcam) and HSPA1B (1:800, Abcam). Horseradish peroxidase-conjugated goat anti-rabbit IgG (1:1500, Cell Signaling Technology) were applied as a secondary antibody for 1 h at room temperature. The immunoreactive bands were detected using an enhanced chemiluminescent detection reagent (Millipore). Signal intensity was measured using a Tanon 5200 Chemiluminescence Imaging System (Tanon, Shanghai, China).

#### Inference of cell-type infiltration based on bulk RNA-seq data

CIBERSORTx is a Web-based tool allowed the creation of the scRNA-seq signature matrix upon which the cell fractions and the cell type-specific gene expression patterns are imputed from bulk RNA-seq data. To establish the proportions of our defined clusters from TCGA CRC bulk RNA-seq data, we used the “FindAllMarkers” function in Seurat to generate a custom reference signature matrix extracted from our scRNA-seq data. Then the Cell Fractions module from CIBERSORTx was applied to perform an estimation of the cell proportions of TCGA CRC samples based on constructed cell-type reference. The “ggplot2” package was employed to visualize comparisons between various cell types in early- and advanced-stage TCGA CRC samples.

#### Survival analysis

The two-sided log-rank test was used to compare Kaplan–Meier survival curves. Survival analyses were carried out by using “survival” (version 3.5–5) and “survminer” (version 0.4.9) R packages.

#### Analysis of TCGA CRC bulk RNA-seq data for mature TLS scores

The mature TLS gene score was calculated by GSVA (version 1.46.0) using the 29-gene TLS signature proposed by Meylan et al. (*IGHA1*, *IGHG1*, *IGHG2*, *IGHG3*, *IGHG4*, *IGHGP*, *IGHM*, *IGKC*, *IGLC1*, *IGLC2*, *IGLC3*, *JCHAIN*, *CD79A*, *FCRL5*, *MZB1*, *SSR4*, *XBP1*, *TRBC2*, *IL7R*, *CXCL12*, *LUM*, *C1QA*, *C7*, *CD52*, *APOE*, *PTLP*, *PTGDS*, *PIM2*, *DERL3*) [19].

## Results

### The single-cell immune landscapes in early- and advanced-stage CRC

**Fig. 1:**
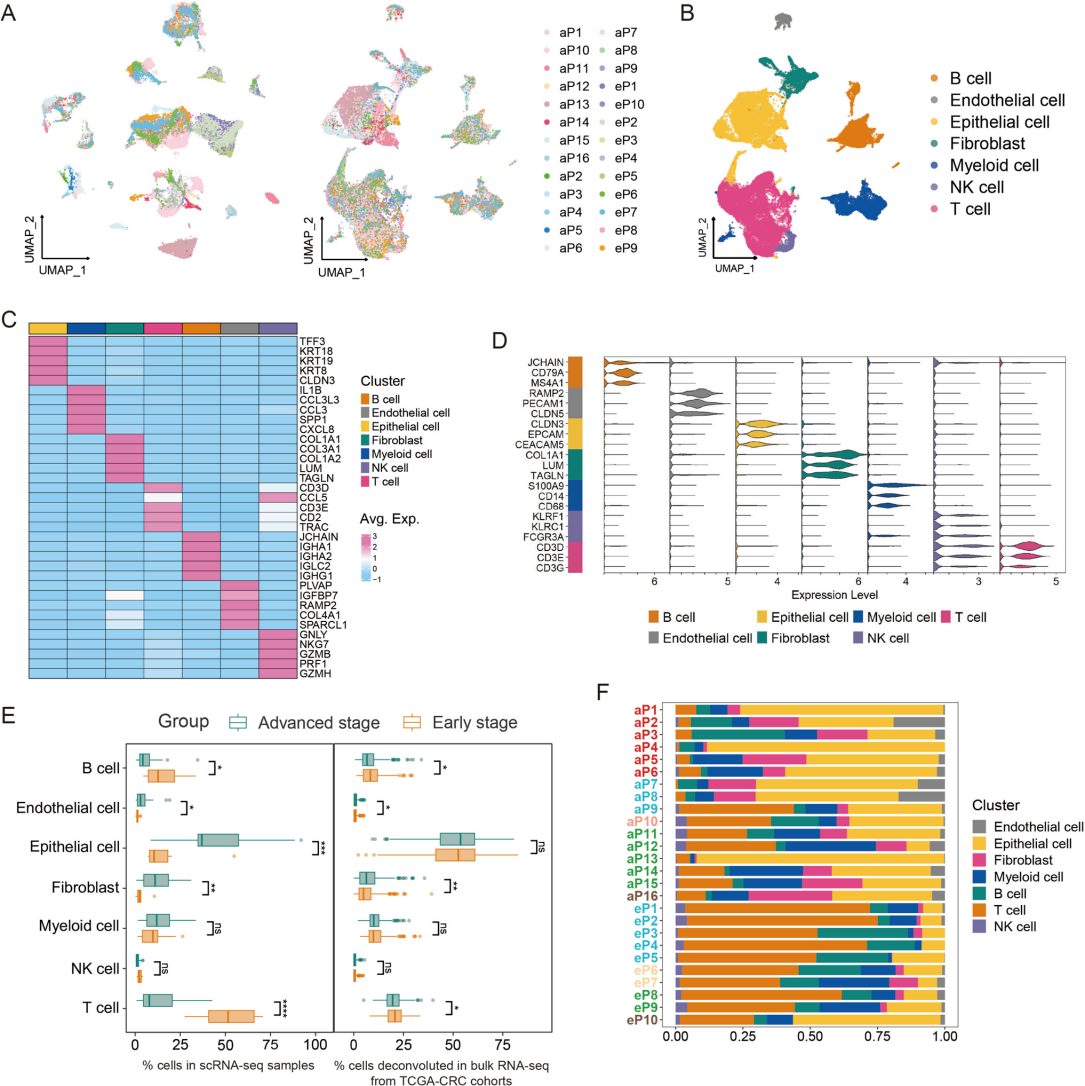
Single-cell landscape of colorectal cancer from early- and advanced-stage CRC patients. **A** UMAP plots of cells from 26 CRC samples before (left) and after (right) the integration analysis of single-cell data from different GEO datasets. **B** UMAP plot showing the seven broad cell types in CRC samples. **C** Heatmap displaying average expression of differentially expressed genes (DEGs). For each cluster, the top five genes and their relative expression levels in all CRC cells are shown. **D** Violin plot displaying the expression of known canonical marker gene across diverse cell types in CRC samples. **E** Box plot showing the fraction of cell types that originated from each patient. Leftmost on the plot corresponds to patient IDs. IDs starting with “e” denote early-stage CRC patients, “a” represents advanced-stage CRC patients, and different colors signify patients from distinct GEO datasets. **F** Box plots showing the cell-type abundance for samples from different patient groups, as measured by scRNA-seq data or deconvoluted bulk RNA-seq from TCGA CRC cohort. P values are calculated by two-sided Wilcoxon test. *p < 0.05; **p < 0.01; ***p < 0.001; ****p < 0.0001; ns, not significant

We included six single-cell RNA sequencing datasets of CRC primary tumors, comprising 10 early-stage and 16 advanced-stage patients without prior neoadjuvant therapy. After rigorous quality control and data integration (Figs. 1A and S1), scRNA-seq data including 22,282 from early-stage CRC and 20,221 from advanced-stage CRC were subjected to further analysis. We performed unsupervised clustering and visualized the results with UMAP, revealing seven cell types in the TME ranked by abundance: T cells, epithelial cells, myeloid cells, B cells, fibroblasts, endothelial cells and natural killer (NK) cells, each with distinct marker signatures (Fig. 1B-D).

The proportions of these cell types in the TME differed significantly between early and advanced CRC except myeloid and NK cells (Fig. 1E). T cells (*p* < 0.001) and B cells (*p* = 0.014) were found to be more abundant in early-stage CRC. Interestingly, this trend was perfectly preserved within each single dataset (GSE200997, GSE144735 and GSE132465) that included both early- and late-stage tumors (Fig. 1F). In contrast, advanced tumor was characterized by a higher proportion of epithelial cells (*p* < 0.001), fibroblasts (*p* = 0.002) and endothelial cells (*p* = 0.041). In bulk RNA-seq analysis, we confirmed the differential distribution of T and B cells (Fig. 1E), implying they play key roles in CRC progression.

### Reduced cytotoxicity and elevated exhaustion markers in immune cells of advanced CRC

**Fig. 2:**
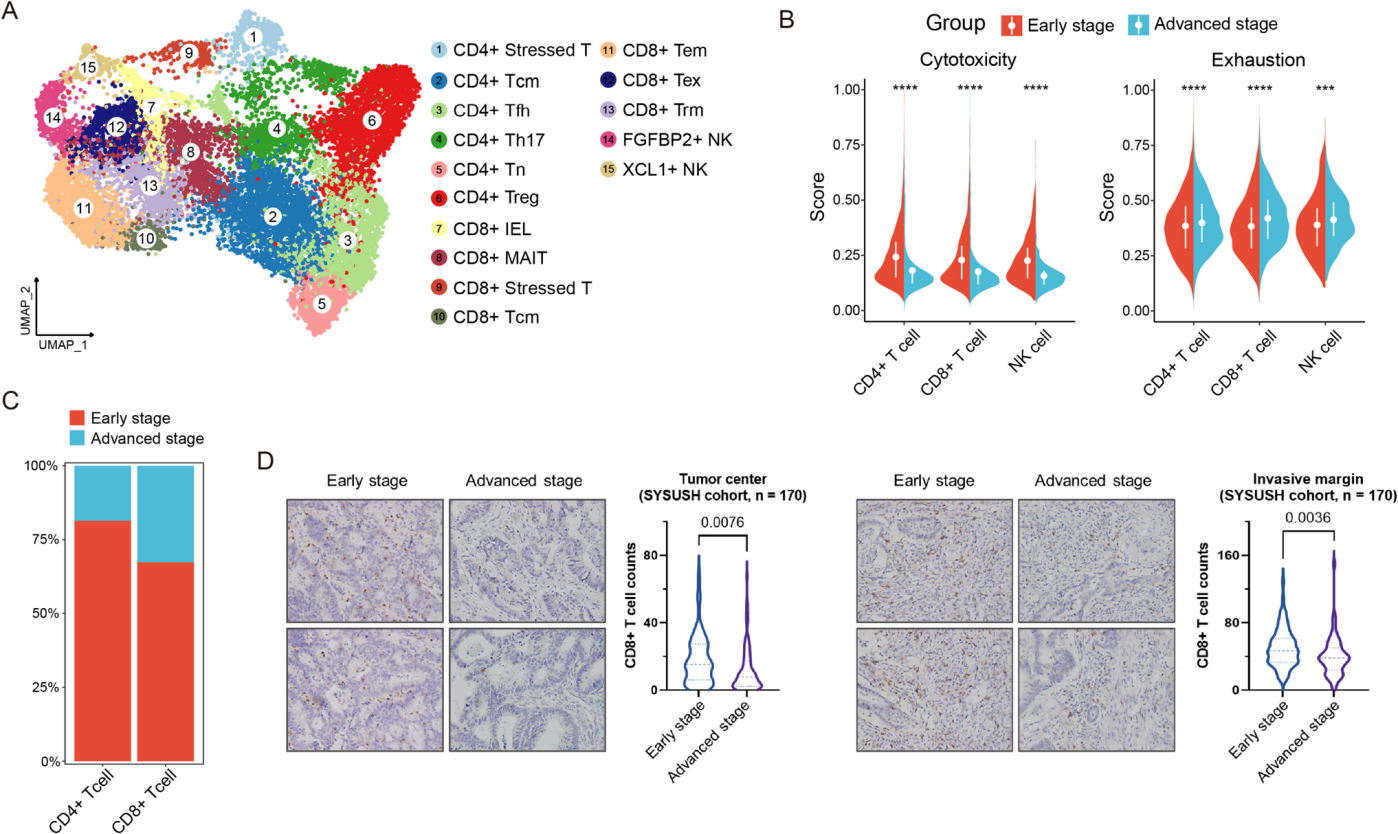
The TME of early-stage CRC exhibits higher effector activity and greater immune cell infiltration. **A** UMAP plots of T and NK cell subsets. **B** Semi-violin plots of the average cytotoxic and exhausted signature scores for CD4 + T, CD8 + T and NK cells in early- and advanced-stage CRC samples. P values are calculated using two-sided Wilcoxon test. **C** Fraction of CD4 + T and CD8 + T cells in early- and advanced-stage CRC. **D** Representative IHC images of CD8 staining in the center (left) or on the margin (right) of early- and advanced-stage CRC from SYSUSH cohort and corresponding quantification data are presented by violin plot showing the differential CD8 + T cell infiltration between two groups. Two-sided *t*-test. ***p < 0.001; ****p < 0.0001

**Fig. 3:**
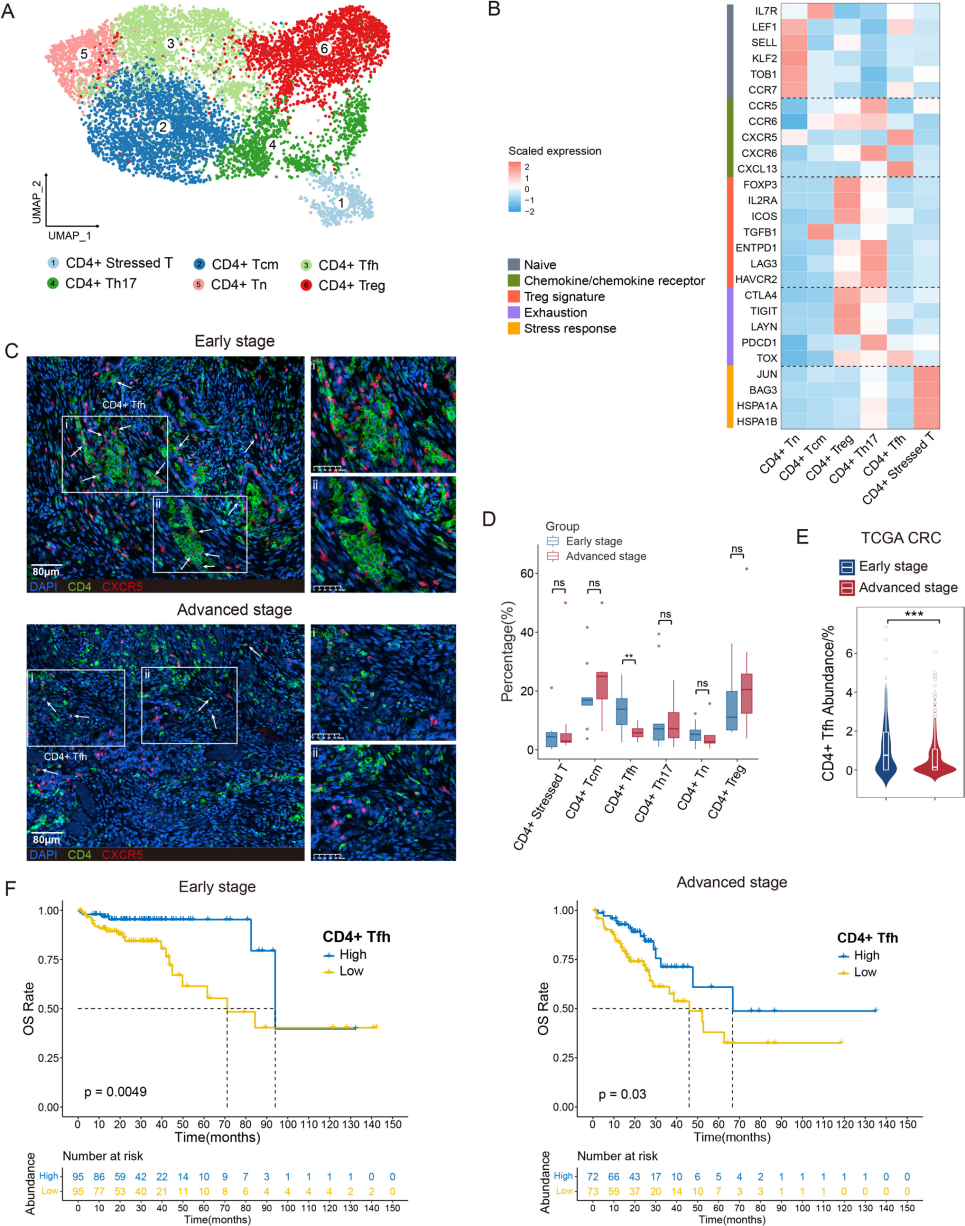
Differences of compositions and functions in CD8 + T cells from early- and advanced-stage CRC. **A** UMAP plot of CD4 + T cell subsets. **B** Heatmap displaying expression of markers associated with naïve/chemokine or chemokine receptor/Treg signature/exhaustion/stress response across CD4 + T cell subsets. **C** mfIHC image of CD4 (green) and CXCR5 (red) in human CRC tissues, counterstained with DAPI (blue). Scale bars: 80 μm. White arrows indicate individual CD4 + Tfh cells. White dashed boxes denote regions enriched with CD4 + Tfh cell clusters, which are magnified in panels i and ii. **D** Box plots showing cellular fractions of each CD4 + T cell subset in CD4 + T cells from early- and advanced-stage CRC samples. **E** Comparison of CD4 + Tfh cell abundance in early- and advanced-stage CRC from TCGA CRC cohort. **F** Kaplan–Meier analysis of overall survival (OS) in early-stage (left) and advanced-stage (right) CRC patients from TCGA CRC cohort, with patients separated by high and low CD4 + Tfh cell abundance in bulk RNA-seq. Survival curves are compared by the log-rank test. P values are calculated using two-sided Wilcoxon test (D and E). **p < 0.01; ***p < 0.001; ns, not significant

**Fig. 4:**
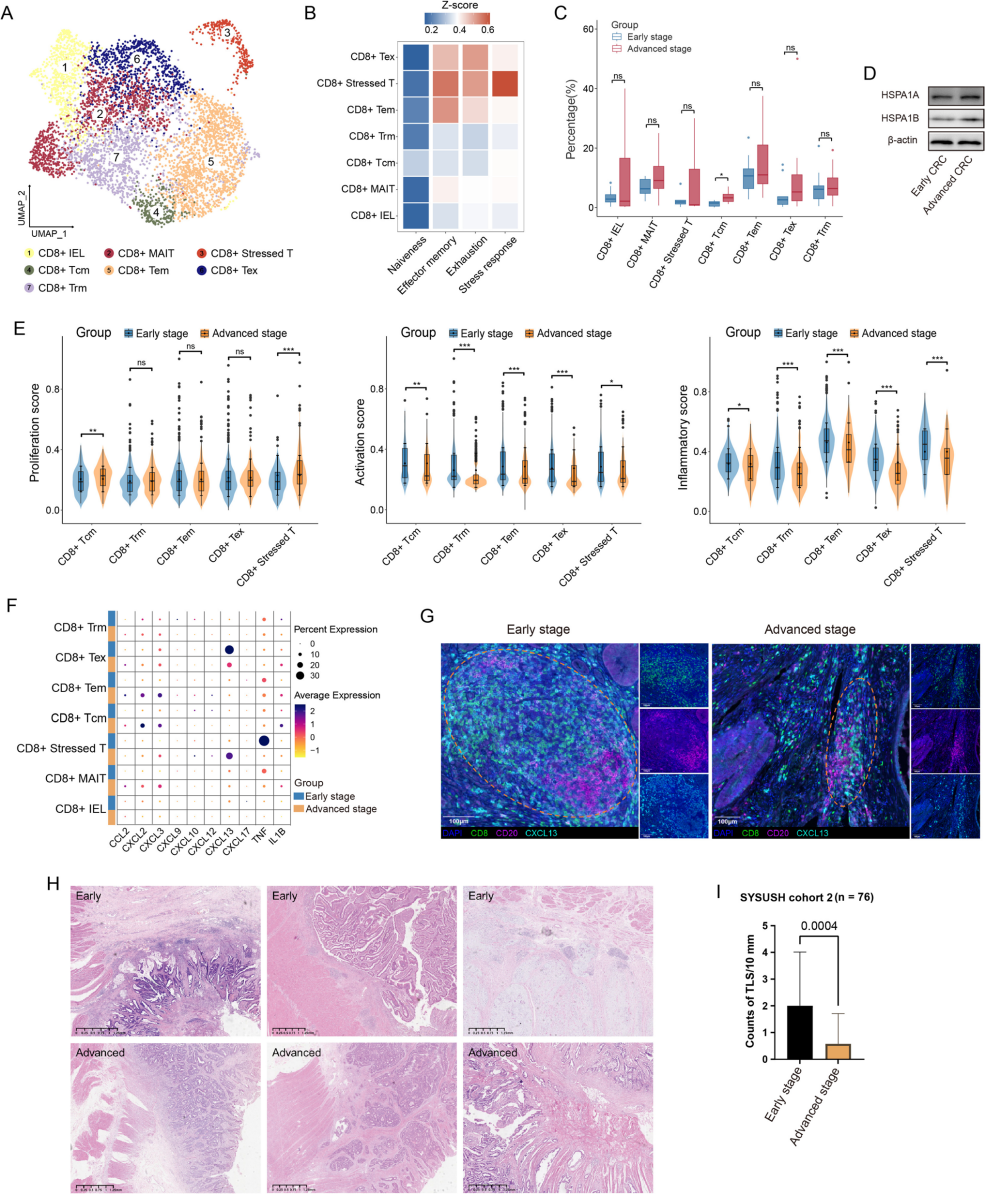
Characteristics of CD8 + T cell subsets and TLSs in early and advanced CRC. A UMAP plot of CD8 + T cell subsets. **B** Heatmap of signature scores of naïve, effector memory and exhaustion among CD8 + T cell subsets. **C** Box plots showing cellular fractions of each CD8 + T cell subset in CD8 + T cells from early- and advanced-stage CRC samples. **D** Western blotting for measuring the protein expressions of HSPA1A and HSPA1B in the tumor tissues from early and advanced CRC. **E** Expression levels of 3 gene signatures across CD8 + T cell subsets from early and advanced CRC tissues. **F** Bubble plot of cytokine and IL-1β expression among CD8 + T cell subsets from early and advanced CRC. **G** Expression of CXCL13 by CD8 + T cells in early- and advanced-stage CRC TME. The TLS structure is characterized by an aggregation of CD20 + B cells and CD8 + T cells. **H** Representative H&E image of TLS in early and advanced CRC. **I** Comparisons of average TLS densities at the invasive front line between early- and advanced-stage CRC from SYSUSH cohort 2. Two-sided *t*-test. P values are calculated by two-sided Wilcoxon test (C and E). *p < 0.05; **p < 0.01; ****p < 0.0001; ns, not significant

Since T and NK cells are key immune effectors influencing anti-tumor activity, we subdivided them into 15 subsets based on known signature genes (Figs. 2A and S2B, S2C, S3A). These included six CD4 + T cell subsets: CD4 + stressed T, characterized by unique expression of stress-related heat shock genes (e.g., *HSPA6*, *HSPH1*) and a stress response gene signature [16, 20], alongside CD4 + Tn (naïve T), CD4 + Tcm (central memory T), CD4 + Tfh (T follicular helper), CD4 + Th17 (type 17 helper T) and CD4 + Treg (regulatory T). Similarly, CD8 + T cell subsets comprised CD8 + stressed T (defined by the same stress-associated transcriptional profile), CD8 + IEL (intraepithelial lymphocyte), CD8 + MAIT (mucosal-associated invariant T), CD8 + Tcm, CD8 + Tem (effector memory T), CD8 + Trm (resident memory T) and CD8 + Tex (exhausted T). Two NK cell subsets (FGFBP2 + NK and XCL1 + NK) were also identified (Figs. 3A, 4A and S2A).

To assess functional potential, we calculated cytotoxicity and exhaustion scores for CD4 + T, CD8 + T and NK cells. Advanced CRC exhibited reduced cytotoxicity scores and elevated exhaustion scores compared to early CRC (Figs. 2B, S3B, S3E; Table S2). These observations suggest a shift toward an immune-suppressive microenvironment in advanced disease, consistent with the hypothesis that early-stage CRC may represent immunologically "hot" tumors. However, functional validation is required to confirm impaired effector function in these cells.

Consistent with reduced immune activation, advanced CRC tumors showed lower abundances of CD4 + and CD8 + T cells (Fig. 2C). In our SYSUSH cohort (*n* = 170), immunohistochemical validation confirmed significantly fewer infiltrating CD8 + T cells in advanced CRC tumor centers (*p* = 0.008) and invasive margins (*p* = 0.004) compared to early-stage tumors (Fig. 2D).

### CD4 + Tfh cells in early CRC shared phenotypic markers with CD4 + GC Tfh cells

To explore the dynamics of immune cell lineages in CRC TME, we first focus on CD4 + T cells. CD4 + Tfh cells were characterized by a gene expression signature critical for Tfh cell function or differentiation, including *CD40LG*, *IL6ST*, *TOX*, *PDCD1* and *CXCR5* (Fig. 3B and Figure S2C) [5, 21, 22]. CD4 + Th17 cells expressed high levels of *CTSH*, *IL17A* and *CXCR6*, while CD4 + stressed T cells were distinguished by elevated expression of heat shock genes and stress response signature (Fig. 3B and Figure S2C). Additionally, CD4 + stressed T cells showed high expression of *CCL5*, *CXCL1* and *CXCL3* in advanced CRC (Figure S2D-S2E), which have been reported to recruit Tregs and macrophages into the TME [23–25]. We also observed a significant decrease of CD4 + Tfh cells in advanced CRC (*p* = 0.004) (Fig. 3D). Using a classic CD4/CXCR5/DAPI mfIHC, we examine CD4 + Tfh cell organization within the CRC TME. As expected, CXCR5 + CD4 + Tfh cells were localized in TLSs [26] and also present in the tumor center and invasive margin, with increased numbers in early CRC (Figs. 3C and S2G).

To investigate the role of CD4 + Tfh cells in the TME, we first conducted survival analyses using the TCGA CRC dataset. More CD4 + Tfh cells were enriched in early CRC TME (Fig. 3E) and higher CD4 + Tfh cell infiltration in TCGA CRC samples was associated with better OS in both early and advanced CRC patients (Fig. 3F). Recent reports indicate that CD4 + Tfh cells are tumor-reactive, which may explain their impact on patient survival [27, 28].

We further performed GO analysis on the DEGs of CD4 + Tfh cells in early and advanced CRC. Interestingly, the DEGs of CD4 + Tfh cells in early tumors were highly enriched in immune-related functions such as lymphocyte activation, differentiation and immune signaling pathways. In contrast, the DEGs enriched in advanced tumors showed weaker relevance to immune responses (Figure S2F). GSEA revealed that CD4 + Tfh cells in early CRC had more pronounced GC Tfh-associated gene expression [17], such as *ICOS*, *BTLA*, *IL21*, *CXCL13*, *CXCR5* and *TIGIT* (Figure S2H; Table S2). Notably, CD4 + GC Tfh cells are essential for GC formation by providing necessary help to GC B cells [29]. Combined, these findings highlighted the critical role of CD4 + Tfh cells in CRC, with early CRC exhibiting CD4 + Tfh cells that adopted a GC Tfh phenotype.

### CD8 + Tex cells might promote TLS generation through CXCL13 secretion in early CRC and lost function during tumor progression

Among CD8 + T cells, we identified seven distinct subsets: CD8 + IEL, CD8 + MAIT, CD8 + Tcm, CD8 + Tem and CD8 + Trm cells; CD8 + Tex and CD8 + stressed T cells, the latter two exhibiting exhaustion features (Fig. 4A and 4B). We assessed the proliferation, activation and inflammatory signatures of CD8 + T cell subsets between early- and advanced-stage CRC. The proliferation signatures of CD8 + stressed T and CD8 + Tcm cells were significantly increased in advanced CRC (Fig. 4E, Table S2), although the proportions of CD8 + T cell subsets remained unchanged (Fig. 4C). Conversely, the activation and inflammatory signatures of the major CD8 + T cell subsets were decreased in advanced CRC (Fig. 4E). Higher baseline levels of CD8 + T cell activation and inflammation have been associated with better responses to ICI therapy in early CRC [30]. Furthermore, CD8 + stressed T cells uniquely expressed stress response features similar to those observed in CD4 + stressed T cells (Figs. 4B and S3A). Trajectory analysis identified CD8 + and CD4 + stressed T cells as terminal states (Figure S3D). Additionally, we found increased levels of HSPA1A and HSPA1B proteins in advanced CRC samples through western blot analysis (Fig. 4D). Recent studies have reported that T cells with high expression of stress-related heat shock genes, particularly *HSPA1A* and *HSPA1B*, are strongly associated with resistance to ICIs [16]. These findings may partially explain the suboptimal efficacy of ICIs in advanced CRC.

CD8 + T cells gradually becoming inactive in the TME may partially differentiate into CD8 + Tex and decide the responsiveness to ICI therapy [31, 32]. We analyzed some marker genes of CD8 + Tex cells and found that they expressed higher levels of effector markers *GZMA*, *GZMK* and *IFNG*, along with reduced levels of exhaustion markers *CTLA4*, *HAVCR2* and *LAG3* in early CRC (Figure S3B). Along pseudotime trajectory 1 of CD8 + T cells, exhaustion signatures were upregulated, while cytotoxicity signatures were downregulated (Figure S3E). These results suggested a transitional process wherein CD8 + Tex cells gradually lost their effector function due to tumor-dependent mechanisms in the TME [33].

Through investigating the cytokines secreted by CD8 + T cells, we found that CXCL13 expression was significantly higher in CD8 + Tex cells in early CRC (Fig. 4F). CXCL13 promotes B cell infiltration into tumors and is crucial for TLS development, with its density serving as a favorable prognostic indicator for immunotherapy response in various cancers [18, 34]. Pathological analysis revealed that the average TLS density at the invasive front line of early CRC was 2.00/10 mm, in contrast to advanced CRC where it was a mere 0.58/10 mm (Fig. 4H–I). We even observed the coexistence of early, primary and secondary TLS in the same microscopic field of early CRC, indicating the extensive formation and maturation process of TLS (Figure S3C). Moreover, CD8 + T cells in early tumors expressed higher levels of CXCL13 (Fig. 4G). These results indicated that CXCL13 expression on CD8 + Tex cells might facilitate TLS formation in early CRC.

### BGC and CD4 + Tfh cells collaboratively promoted TLS maturation in early CRC

**Fig. 5:**
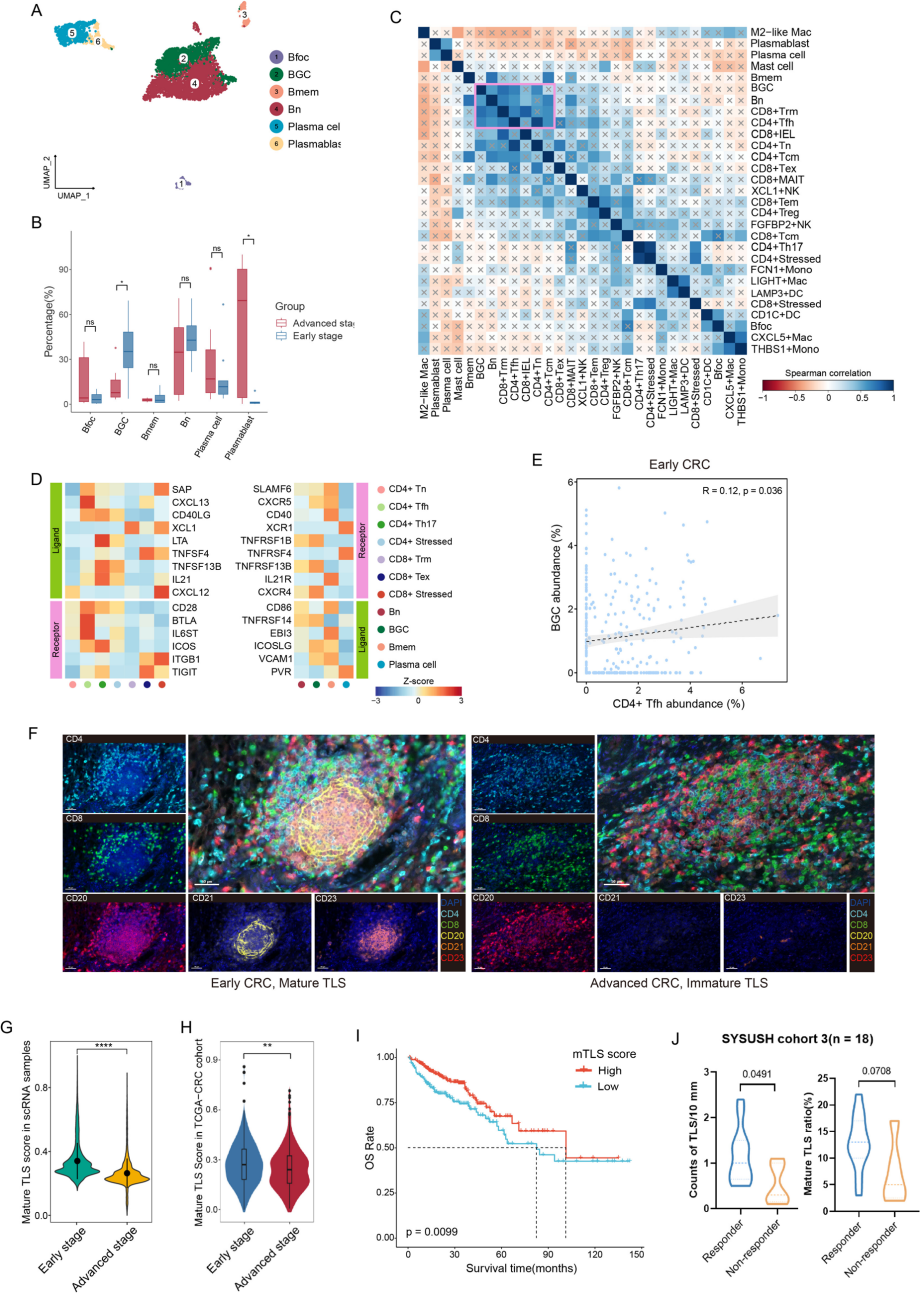
BGC and CD4 + Tfh cells concertedly expand and more mature TLSs are observed in early CRC. A UMAP plots of B cells colored and labeled by subsets. **B** Box plots comparing the cellular proportions of each subset of B cells between early- and advanced-stage CRC. P values are calculated using two-sided Wilcoxon test. **C** Correlation heatmap of immune cell frequencies in CRC. x represents no correlation between the two corresponding cell types. **D** Heatmap showing the expression of ligand–receptor pairs highly expressed in B and T cells. **E** Scatterplots showing the Spearman correlation of CD4 + Tfh cell abundances with BGC cell abundances of early CRC in TCGA CRC data. **F** Representative mfIHC images of mature (left) and immature TLS (right) from early and advanced CRC, respectively. Scale bars, 50 μm. **G-H** Violin plots comparing mature TLS score distributions between early- and advanced-stage CRC in single-cell sequencing data (G) and TCGA CRC data (H). **I** Kaplan–Meier plots of OS among patients with high and low mTLS score in TCGA CRC cohort. Survival curves were compared by the log-rank test. **J** Comparisons of average TLS densities (left) and mature TLS ratios (right) at the invasive front line between responders and non-responders for neoadjuvant anti-PD-1 therapy from SYSUSH cohort 3. *p < 0.05; **p < 0.01; ****p < 0.0001; ns, not significant

To understand the variability in TLS formation between early and advanced stages, we analyzed the heterogeneity of B cells, the primary cell types in TLS. B cells were partitioned into six subsets, each with its unique signature genes (Figs. 5A and S4A). By comparing transcriptomic profiles of B cells between early- and advanced-stage CRC, we observed that B cells in early-stage CRC predominantly expressed genes associated with antigen processing and presentation, type II interferon response and T cell activation. In contrast, B cells in advanced CRC showed a higher expression of genes linked to protein quality control, apoptotic signaling and stress response pathways (Figure S4B). Thus, the function of B cells in the early CRC TME might be mainly attributed to activating T cells.

BGC cells marked by high *CXCR4*, *CD69*, *NR4A1* and *NR4A2* gene expression [5] (Figure S4A) were significantly increased in early CRC (*p* = 0.028). Given BGC cells and CD4 + Tfh cells are essential components of TLS and functionally interact with each other [35], we analyzed lineage-normalized cell-type frequencies to explore these interactions within the tumor microenvironment. As expected, BGC cells showed strong correlations with CD4 + Tn and CD4 + Tfh cells (Fig. 5C). Further analysis of ligand–receptor pairs revealed that CXCR5 was highly expressed in the BGC subset. Several ligand–receptor pairs involved in B cell differentiation and recruitment were significantly expressed between BGC and CD4 + Tfh cells, including *SAP*-*SLAMF6*, *CXCL13*-*CXCR5*, *TNFRSF14*-*BTLA*, *ICOSLG*-*ICOS* and *CXCL12*-*CXCR4* (Fig. 5D). Indeed, mfIHC analysis showed CD4 + Tfh cells were adjacent to the B cell zone, suggesting that CD4 + Tfh cells interacted with B cells directly (Figure S4C). The high abundance of BGC cells in early-stage CRC and their interaction with CD4 + Tfh cells indicates the potential formation of TLS.

We used bulk RNA sequencing data from CRC samples in the TCGA dataset to validate the connection between CD4 + Tfh and BGC cells. The abundance of CD4 + Tfh cells showed a strong correlation with BGC cell abundance in early-stage CRC but not in advanced-stage CRC (Figs. 5E and S4D). Using the CellChat algorithm, we further revealed that the cellular interaction strength between CD4 + Tfh cells and BGC cells is reduced in advanced CRC compared to early CRC (Figure S4E). Notably, a stronger *CD40*–*CD40LG* interaction between BGC and CD4 + Tfh cells was observed in early CRC (Figure S4F and S4G).

We also identified a higher prevalence of mature TLSs in the early CRC TME (Fig. 5F). Mature TLSs defined by the presence of a germinal center with B cells, follicular dendritic cells surrounding by a T cell zone, rather than immature TLSs, are associated with clinical benefits in various cancer type [36–39]. In this study, we were surprised to find that TLS in early-stage CRC exhibited significantly higher maturity scores [38] (Fig. 5G), a result that was also confirmed in the TCGA CRC cohort (Fig. 5H). Moreover, higher mTLS scores were found to be associated with improved survival (Fig. 5I). As expected, the proportion of elevated mTLS scores was significantly higher in early CRC compared to advanced CRC (early CRC: 210/308 and advanced CRC: 143/248 respectively, *p* = 0.013). Additionally, in our institutional cohort, we found that in advanced rectal cancer patients treated with anti-PD-1 therapy, responders exhibited a higher density of TLS infiltration compared to non-responders, with a trend toward a greater proportion of mature TLS (Fig. 5J, Table S3). These findings confirmed the clinical benefits of mature TLSs in CRC and suggested that the interactions between CD4 + Tfh and BGC cells might differ between early and advanced CRC, potentially influencing the observed variations in TLS formation across disease stages.

## Discussion

Discrepancies exist in the response to ICI therapy between early and advanced CRC. Here, we discovered significant disparities in immune cell subpopulations and the status of TLSs in early and advanced CRC without neoadjuvant therapy. These variances reflect dynamic changes in the TME during tumor progression, which may be related to the response to ICI therapy.

We observed that early and advanced tumors share the same immune cell composition, yet exhibit substantial differences in their proportions and functionalities. Our previous research indicated that MSI CRC is often classified as “hot tumors,” making them more responsive to ICI therapy [8]. In this study, early CRC demonstrated overall higher infiltration of various immune cells, along with increased cytotoxic activity within these populations. These findings suggest that, beyond microsatellite status [40] and tumor mutation burden [41], the progression of CRC may also affect the response to immunotherapy, consistent with previous reports [1].

Tumor-specific CD8 + T cells are key effector cells in anti-tumor immune responses, with their differentiation status closely linked to ICI therapy effectiveness [42]. Unlike most studies focusing on the tumor core, we found that the abundance of CD8 + T cells in the invasive margin, a specific distribution area of TLS, is significantly reduced in advanced CRC. Additionally, our analysis revealed that early-stage CD8 + Tex cells exhibited higher effector function compared to late-stage CD8 + Tex cells, consistent with previous studies [42, 43]. Further results indicated that the expression level of CXCL13 was higher in the CD8 + Tex subset in early CRC, which was associated with B cell recruitment and TLS formation [44, 45].

The presence of TLS is associated with better prognosis and improved response to immunotherapy [34, 46]. We were intrigued to discover that early-stage CRC exhibited a greater number of TLSs at the tumor invasive front, with higher levels of maturity. In our cohort, we found that both TLS density and maturity may be key factors affecting the response to ICI therapy, although this was observed only in patients with advanced rectal cancer. Despite their recognized importance, the mechanisms driving TLS formation in cancer tissues and their role in intratumoral immune responses are not yet fully understood.

Further investigation into the differences in TLS formation between early and late stages revealed that T cell and B cell interactions were a critical factor. We utilized mfIHC analysis to demonstrate the enrichment of CXCR5 + CD4 + Tfh cells within TLS in early CRC, indicating the critical role of CD4 + Tfh cells in TLS. Furthermore, we confirmed a strong correlation in both the abundance and spatial distribution of CD4 + Tfh cells and BGC cells in early CRC, both of which are key components of GC, a hallmark of mature TLS [35, 47]. Meanwhile, our findings indicated that the strength of the CD40–CD40L receptor–ligand interaction between BGC and CD4 + Tfh cells was significantly higher in early CRC. Previous research has demonstrated that the CD40L expressed on the surface of activated CD4 + T cells binds to CD40 on BGC, promoting B cell proliferation and affinity maturation, while further activating T cells and facilitating the formation of GC in TLS [48, 49]. The above results indicated that in the invasive margin of early CRC, activated CD8 + Tex cells facilitated the spatial accumulation of a substantial number of CD4 + Tfh cells and BGC cells by secreting chemokine CXCL13, which enhanced the CD40–CD40L interaction and ultimately promoted the formation and maturation of germinal centers within TLS. However, this process was significantly diminished in advanced CRC, which may contribute to the poor response to ICI therapy.

This study has several limitations. Batch effects were introduced by using six publicly available datasets from various sources worldwide; however, this approach also helped minimize the sampling bias within single study. In addition, the CD40–CD40L interaction between CD4 + Tfh cells and BGC cells is essential for TLS maturation in the invasive margin of early CRC, and further molecular experiments are needed to elucidate the reasons for its attenuation in advanced stages. Due to the limited sample size of the SYSUSH cohort 3, we only validated the impact of TLS abundance and maturity on ICI therapy in advanced rectal cancer, without excluding the potential interference of neoadjuvant chemotherapy and targeted therapies administered to certain patients. Larger and more rigorous patient cohorts are required to enhance the robustness of the conclusions drawn from this study.

## Conclusions

In conclusion, our study globally analyzed the major immune components of early- and advanced-stage CRC without considering microsatellite status, revealing significant discrepancies related to tumor immunity. These variances may be associated with TLS formation and impact the response to immunotherapy. These findings contribute to understanding the dynamic changes during tumor progression and offer new perspectives for the treatment of advanced-stage CRC.

## Supplementary Information

Below is the link to the electronic supplementary material.

Supplementary file1 (TIF 28173 KB)

Supplementary file2 (TIF 43206 KB)

Supplementary file3 (TIF 35081 KB)

Supplementary file4 (TIF 58209 KB)

Supplementary file5 (XLSX 1862 KB)

Supplementary file6 (XLSX 17 KB)

Supplementary file7 (XLSX 11 KB)

## Data Availability

All relevant data not presented in the main figures or in the supplementary data are available from the authors.
