# Supplementary figures and images for: Dynamics of tertiary lymphoid structures and immune cross talk in early versus advanced colorectal cancer: potential implications for immunotherapy
Source: Cancer Immunol Immunother. 2025 Apr 26;74(6):185. doi: 10.1007/s00262-025-04027-x (PMC12033131; doi:10.1007/s00262-025-04027-x)

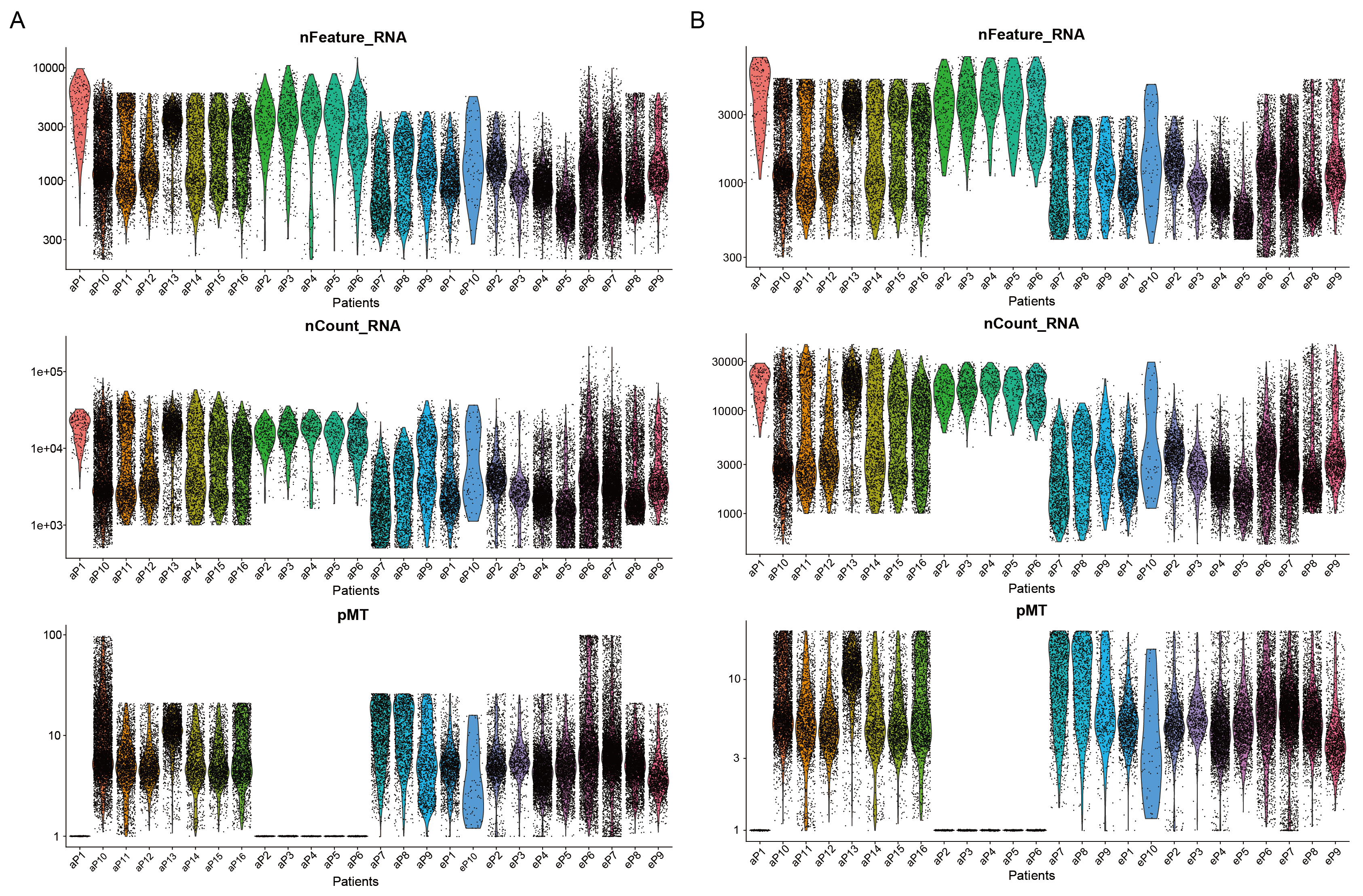

Supplement: Supplementary file 1 — Supplementary file1 (TIF 28173 KB) [file 262_2025_4027_MOESM1_ESM.tif]

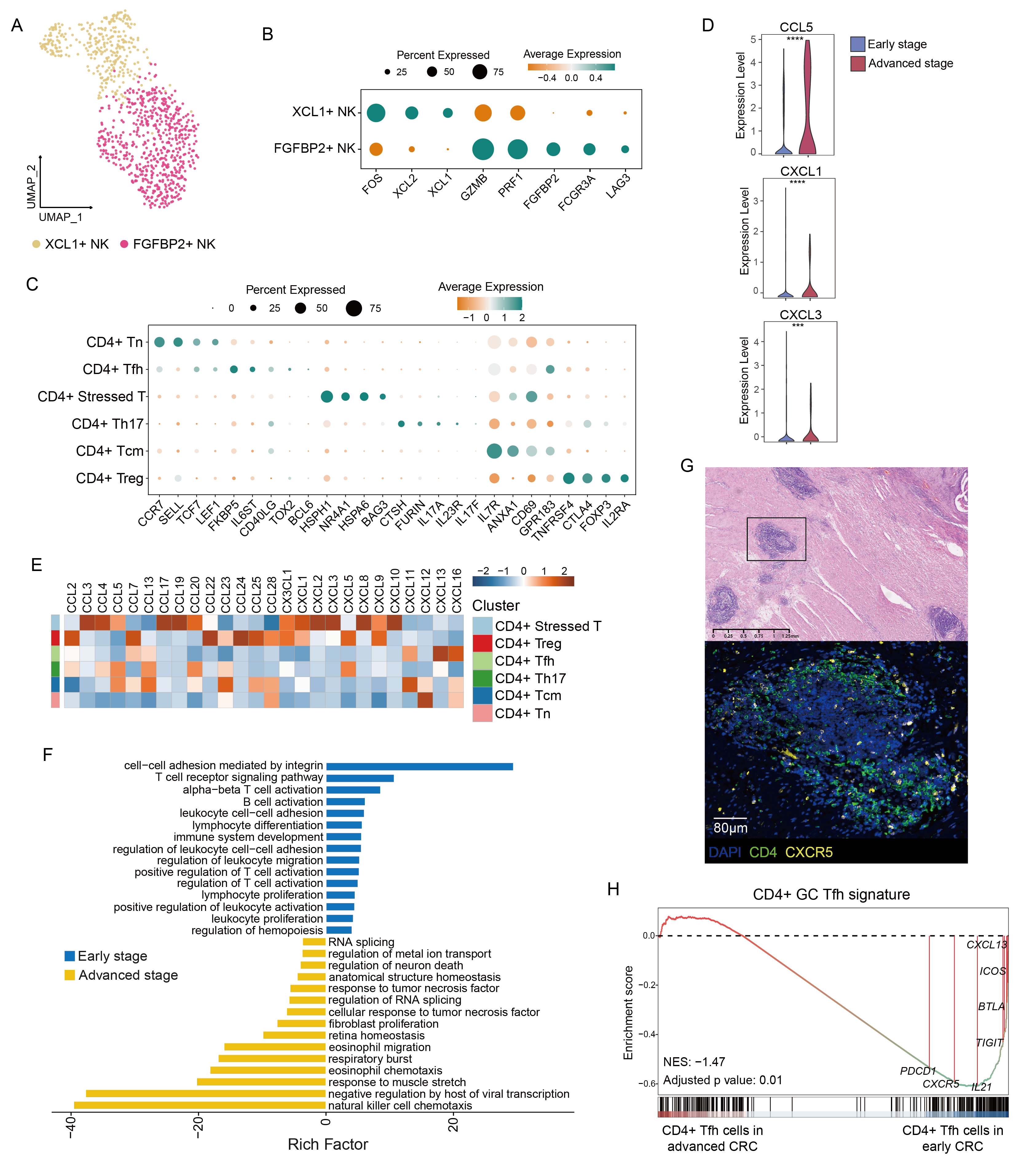

Supplement: Supplementary file 2 — Supplementary file2 (TIF 43206 KB) [file 262_2025_4027_MOESM2_ESM.tif]

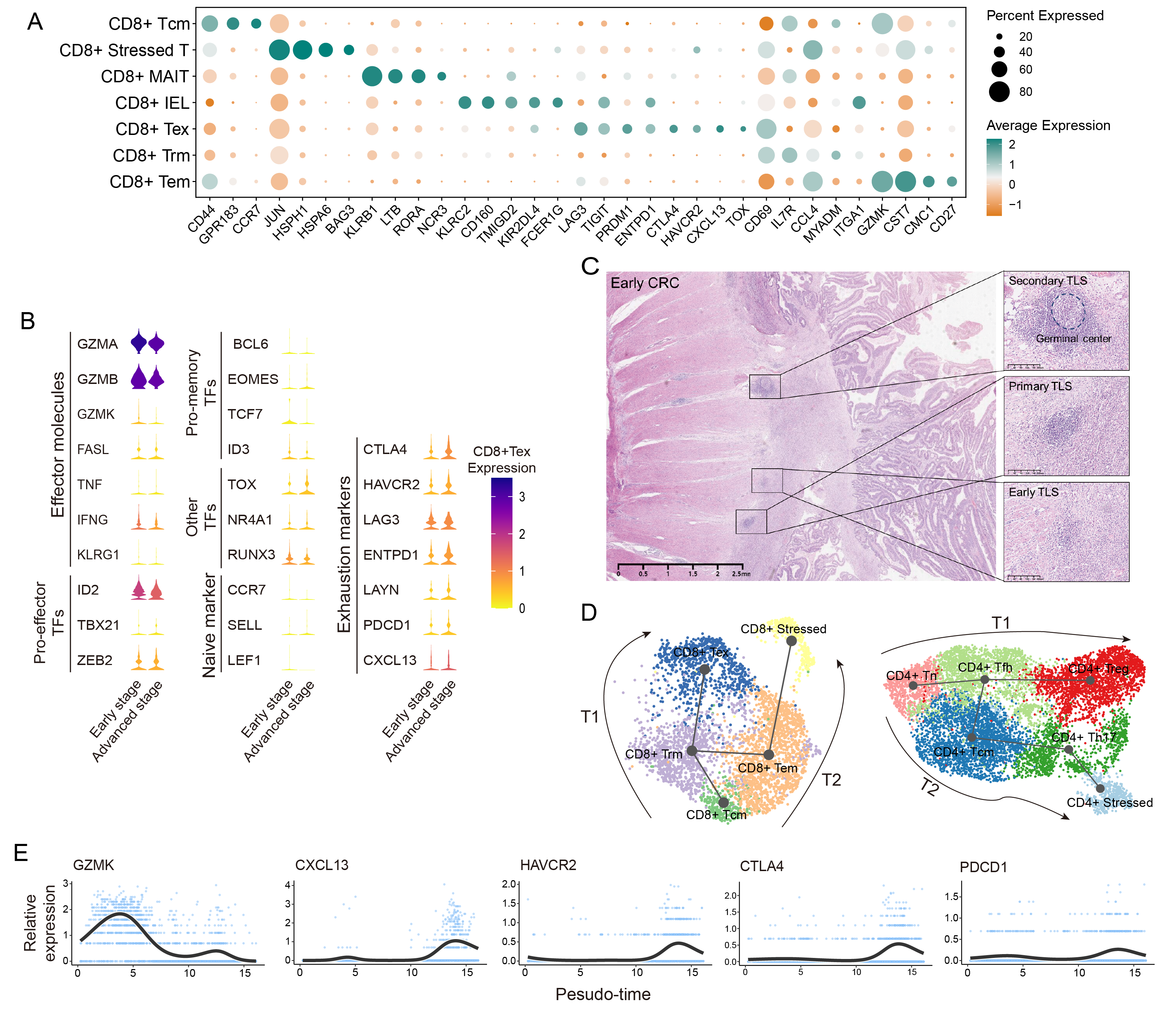

Supplement: Supplementary file 3 — Supplementary file3 (TIF 35081 KB) [file 262_2025_4027_MOESM3_ESM.tif]

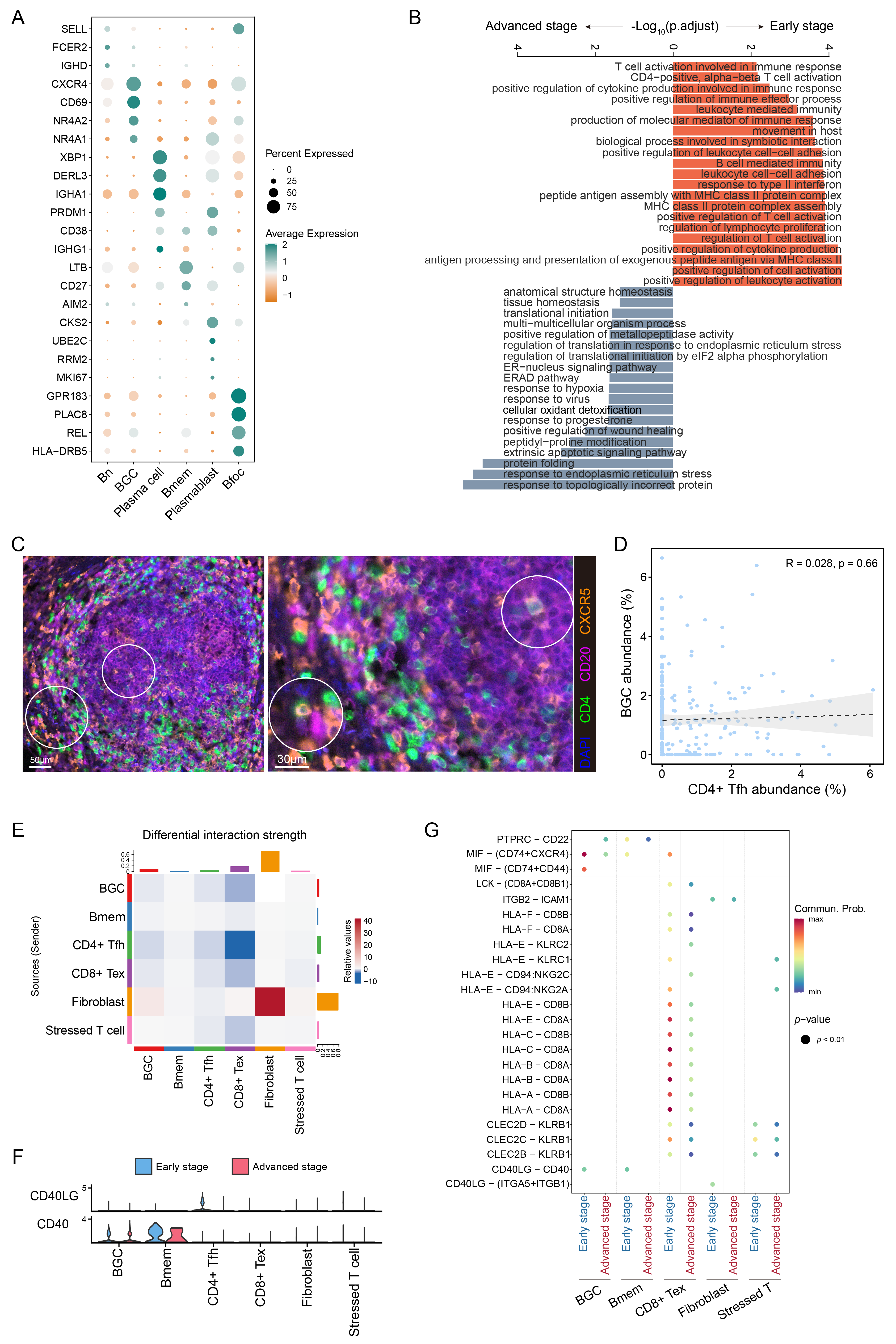

Supplement: Supplementary file 4 — Supplementary file4 (TIF 58209 KB) [file 262_2025_4027_MOESM4_ESM.tif]
